# Intravenous fluid therapy in the perioperative and critical care setting: Executive summary of the International Fluid Academy (IFA)

**DOI:** 10.1186/s13613-020-00679-3

**Published:** 2020-05-24

**Authors:** Manu L. N. G. Malbrain, Thomas Langer, Djillali Annane, Luciano Gattinoni, Paul Elbers, Robert G. Hahn, Inneke De laet, Andrea Minini, Adrian Wong, Can Ince, David Muckart, Monty Mythen, Pietro Caironi, Niels Van Regenmortel

**Affiliations:** 1 Department of Intensive Care Medicine, University Hospital Brussels (UZB), Laarbeeklaan 101, 1090 Jette, Belgium; 2 Faculty of Medicine and Pharmacy, Vrije Universiteit Brussel (VUB), Laarbeeklaan 103, Jette, 1090 Belgium; 3 International Fluid Academy, Lovenjoel, Belgium; 4 School of Medicine and Surgery, Milano-Bicocca University, Milan, Italy; 5 Department of Anesthesia and Critical Care, ASST Grande Ospedale Metropolitano Niguarda, Milan, Italy; 6 General Intensive Care Unit, Raymond Poincaré Hospital (GHU APHP Université Paris Saclay), U1173 Inflammation & Infection, School of Medicine Simone Veil, UVSQ-University Paris Saclay, 104 Boulevard Raymond Poincaré, 92380 Garches, France; 7 Emergency and Intensive Care Medicine, University of Göttingen, Göttingen, Germany; 8 Department of Intensive Care Medicine, Amsterdam UMC, Location VUmc, Amsterdam, The Netherlands; 9 Karolinska Institutet at Danderyds Hospital (KIDS), Stockholm, Sweden; 10 Department of Intensive Care Medicine, Ziekenhuis Netwerk Antwerpen, ZNA Stuivenberg, Antwerp, Belgium; 11 Department of Intensive Care Medicine and Anaesthesia, King’s College Hospital, Denmark Hill, London, UK; 12 Department of Intensive Care Medicine, Laboratory of Translational Intensive Care Medicine, Erasmus MC, University Medical Center Rotterdam, Rotterdam, The Netherlands; 13 Department of Surgery, Nelson R Mandela School of Medicine, University of KwaZulu-Natal, Durban, South Africa; 14 Level I Trauma Unit and Trauma Intensive Care Unit, Inkosi Albert Luthuli Central Hospital, Durban, South Africa; 15 University College London Hospitals, National Institute of Health Research Biomedical Research Centre, London, UK; 16 SCDU Anestesia e Rianimazione, Azienda Ospedaliero-Universitaria S. Luigi Gonzaga, Orbassano, Italy; 17 Dipartimento di Oncologia, Università degli Studi di Torino, Turin, Italy; 18 Department of Intensive Care Medicine, Ziekenhuis Netwerk Antwerpen, ZNA Stuivenberg, Antwerp, Belgium

**Keywords:** Fluid therapy, Intensive care units, Resuscitation, Maintenance, Water–electrolyte balance, Goal-directed, Crystalloids, Acid base, Sodium, Chloride

## Abstract

Intravenous fluid administration should be considered as any other pharmacological prescription. There are three main indications: resuscitation, replacement, and maintenance. Moreover, the impact of fluid administration as drug diluent or to preserve catheter patency, i.e., fluid creep, should also be considered. As for antibiotics, intravenous fluid administration should follow the four Ds: drug, dosing, duration, de-escalation. Among crystalloids, balanced solutions limit acid–base alterations and chloride load and should be preferred, as this likely prevents renal dysfunction. Among colloids, albumin, the only available natural colloid, may have beneficial effects. The last decade has seen growing interest in the potential harms related to fluid overloading. In the perioperative setting, appropriate fluid management that maintains adequate organ perfusion while limiting fluid administration should represent the standard of care. Protocols including a restrictive continuous fluid administration alongside bolus administration to achieve hemodynamic targets have been proposed. A similar approach should be considered also for critically ill patients, in whom increased endothelial permeability makes this strategy more relevant. Active de-escalation protocols may be necessary in a later phase. The R.O.S.E. conceptual model (Resuscitation, Optimization, Stabilization, Evacuation) summarizes accurately a dynamic approach to fluid therapy, maximizing benefits and minimizing harms. Even in specific categories of critically ill patients, i.e., with trauma or burns, fluid therapy should be carefully applied, considering the importance of their specific aims; maintaining peripheral oxygen delivery, while avoiding the consequences of fluid overload.

## Introduction

Intravenous fluids have been in clinical use for over a century, yet the medical and scientific community have only recently begun to appreciate the importance of judicious fluid administration, the necessity to handle them as any other drug we prescribe [1–4], and the considerable side effects with which they may be associated [5, 6].

Three major indications exist for intravenous fluid administration [1, 4, 7–9]: resuscitation, replacement, and maintenance. *Resuscitation fluids* are used to correct an intravascular volume deficit or acute hypovolemia; *replacement solutions* are prescribed to correct existing or developing deficits that cannot be compensated by oral intake alone [6]; *maintenance solutions* are indicated in hemodynamically stable patients that are not able/allowed to drink water in order to cover their daily requirements of water and electrolytes [10, 11]. In addition to these classical indications, the quantitative relevance of fluids administered as drug diluents and to guarantee catheter patency, the so-called *fluid creep*, has been recently underlined [12, 13].

Although the use of intravenous fluids is one of the most common interventions in medicine, the ideal fluid does not exist. In light of recent evidence, a reappraisal of how intravenous fluids should be used in the perioperative and critical care setting is warranted. Here, we present the executive summary on this area of the International Fluid Academy (https://www.fluidacademy.org).

## The four Ds of fluid management

Similarly to antibiotics, the 4 Ds of fluid therapy need to be considered (Table 1) [4].

**Table 1:** Analogy between the 4 Ds of antibiotic and fluid therapy Stewardship. Adapted from Malbrain M.L.N.G. et al. [4] with permission

	Description	Antibiotics	Fluids
Drug	Inappropriate therapy	More organ failure, longer ICU/hospital length of stay, longer duration mechanical ventilation (MV)	Hyperchloremic metabolic acidosis, more acute kidney injury, more need for renal replacement therapy, increased mortality
	Appropriate therapy	Key factor in empiric AB choice is consideration of patient risk factors (prior AB use, duration of mechanical ventilation, corticosteroids, recent hospitalization, residence in nursing home, etc.)	Key factor in empiric fluid therapy is consideration of patient risk factors (fluid balance, fluid overload, capillary leak, source control, kidney function, organ function). Do not use glucose as a resuscitation fluid
	Combination therapy	Possible benefits: broader spectrum, synergy, avoidance of emergency of resistance, less toxicity	Possible benefits: specific fluids for different indications (replacement vs maintenance vs resuscitation), less toxicity
	Appropriate timing	Survival decreases with 7% per hour delay. Needs discipline and practical organization	In refractory shock early goal-directed therapy (EGDT) has proven beneficial. The longer the delay, the more microcirculatory hypoperfusion
Dosing	Pharmacokinetics	Depends on distribution volume, clearance (kidney and liver function), albumin level, tissue penetration	Depends on type of fluid: glucose remains 10% intravascular, crystalloids 25%, vs colloids 100% after 1 h, and other factors (distribution volume, osmolality, oncoticity, kidney function)
	Pharmacodynamics	Reflected by the minimal inhibitory concentration. Reflected by “kill” characteristics, time (*T* > MIC) vs concentration (*C*_max_/MIC) dependent	Depends on type of fluid and where you want them to go: intravascular (resuscitation), interstitial vs intracellular (cellular dehydration)
	Toxicity	Some ABs are toxic for kidneys, advice on dose adjustment needed. However, not getting infection under control is not helping the kidneys either	Some fluids (HES—starches) are toxic for the kidneys. However, not getting shock under control is not helping the kidneys either
Duration	Appropriate duration	No strong evidence but trend toward shorter duration. Do not use ABs to treat fever, CRP, infiltrates, but use ABs to treat infections	No strong evidence but trend toward shorter duration. Do not use fluids to treat low central venous or mean arterial pressure, urine output, but use fluids to treat hypovolemia
	Treat to response	Stop ABs when signs and symptoms of active infection resolve. Future role for biomarkers (PCT)	Fluids can be stopped when shock is resolved (normal lactate). Future role for biomarkers (NGAL, cystatin C, citrullin, L-FABP)
De-escalation	Monitoring	Take cultures first and have the guts to change a winning team	After stabilization with early adequate fluid management (normal PPV, normal cardiac output, normal lactate), stop ongoing resuscitation and move to conservative late fluid management and late goal-directed fluid removal (= deresuscitation)

*AB* antibiotic, *C*_max_ maximal peak concentration, *CRP* C reactive protein, *EGDT* early goal-directed therapy, *HES* hydroxyl-ethyl starch, *L-FABP* L-type fatty acid-binding protein, *MIC* mean inhibitory concentration, *MV* mechanical ventilation, *NGAL* neutrophil gelatinase-associated lipocalin, *PCT* procalcitonin, *PPV* pulse pressure variation

### Drug

Fluids are drugs with indications, contraindications, and side effects. Different indications need different types of fluids, e.g., *resuscitation* fluids should focus on rapid restoration of circulating volume; *replacement* fluids must mimic the fluid that has been lost; *maintenance fluids* must deliver basic electrolytes and glucose for metabolic needs.

### Dosing

The dose makes the poison, as stated by Paracelsus. However, timing and administration rate are equally important for fluids [14, 15]. Of note, in contrast to most drugs, there is no standard therapeutic dose for fluids.

### Duration

The duration of fluid therapy is crucial and volume must be tapered when shock is resolved. However, while “starting triggers” for fluid resuscitation are quite clear, clinicians are less aware of “stopping triggers” of fluid resuscitation.

### De-escalation

The final step in fluid therapy is to withhold/withdraw fluids when they are no longer required, thus reducing the risk of fluid overload and related deleterious effects [16].

## Balanced solutions

### The basics

Intravenous “balanced” solutions include crystalloids and colloids with minimal effect on the homeostasis of the *extracellular* compartment, and in particular on acid–base equilibrium and electrolyte concentrations [3]. In addition, the term “balanced” has been recently applied also to fluids with a low chloride content (Cl^−^). Therefore, there are two main categories of balanced solutions (Table 2): (1) fluids causing a minimal effect on acid–base equilibrium, having an electrolyte content with an in vivo strong ion difference (SID), i.e., the SID after metabolism of the organic anion, close to 24–29 mEq/L; (2) fluids having a normal or sub-normal Cl^−^ content (Cl^−^ ≤ 110 mEq/L).

**Table 2:** Electrolyte composition of the main balanced solutions available for intravenous administration. Adapted from Langer et al. [21] with permission

	Crystalloids						Gelatins		Starches	
	Lactated Ringer’s	Acetated Ringer’s	Hartmann’s	PlasmaLyte	Sterofundin ISO^a^	ELO-MEL isoton	Isoplex	Gelaspan	Hextend	Tetraspan
Na^+^ [mEq/L]	130	132	131	140	145	140	145	151	143	140
K^+^ [mEq/L]	4	4	5	5	4	5	4	4	3	4
Ca^2+^ [mEq/L]	3	3	4	–	5	5	–	2	5	5
Mg^2+^ [mEq/L]	–	–	3	3	2	3	1.8	2	0.9	2
Cl^−^ [mEq/L]	109	110	111	98	127	108	105	103	124	118
Lactate [mEq/L]	28	–	29	–	–	–	25	–	28	–
Acetate [mEq/L]	–	29	–	27	24	45	–	24	–	24
Malate [mEq/L]	–	–	–	–	5	–	–	–	–	5
Gluconate [mEq/L]	–	–	–	23	–	–	–	–	–	–
Dextrose [g L-1]	–	–	–	–	–	–	–	–	–	–
Gelatin [g/L]	–	–	–	–	–	–	40	40	–	–
HES [g/L]	–	–	–	–	–	–	–	–	60	60
Dextran [g/L]	–	–	–	–	–	–	–	–	–	–
In-vivo SID [mEq/L]	28	29	29	50	29	45	45.8	56	28	29^b^
Osmolarity [mOsm/L]	278	277	279	294	309	302	284	284	307	297

In-vivo SID—all organic molecules contained in balanced solutions are strong anions. The resulting calculated SID (in vitro-SID) is equal to 0 mEq/L, due to electrical neutrality. Once infused, the organic molecules are metabolized to CO_2_ and water; the resulting in vivo-SID corresponds to the amount of organic anions metabolized

^a^Sterofundin-ISO or Ringerfundin

^b^In vivo-SID of Tetraspan reported in the Table results from the sum of organic anions; of note, there is a discrepancy as compared to the SID calculated as the difference between inorganic cations and inorganic anions (29 mEq/L vs. 33 mEq/L). No clear explanation has been reported from the seller

According to the quantitative approach to acid–base equilibrium [17, 18], the three variables regulating the pH of biologic fluids independently are (1) partial pressure of carbon dioxide (PCO_2_); (2) the concentration of non-volatile weak acids (*A*_TOT_); (3) the strong ion difference (SID), defined as the difference between the sum of all strong cations and the sum of all strong anions [19]. These principles clearly suggest that intravenous fluids may affect pH due to (i) the specific electrolyte content characterizing the solution, therefore altering the SID of the extracellular compartment and (ii) the dilution effect due to the volume infused, thus reducing the concentration of *A*_TOT_ [20–22]. Ideally, the fluid able to leave plasma pH unchanged after its administration, at constant PCO_2_, should balance these variations. Recent studies clearly showed that, in this regard, the ideal balanced solution should have an in vivo SID equal to the baseline concentration of HCO_3_^−^ [23]. If the SID of the infused fluid is greater than plasma HCO_3_^−^, plasma pH will tend toward alkalosis; if the SID of the infused fluid is lower than plasma HCO_3_^−^, plasma pH will tend toward acidosis, as it is always the case for NaCl 0.9%, the so-called “normal” saline [24].

As stated above, the definition of “balanced” solution includes also a category of iso- and near-isotonic fluids with a low Cl^−^ content (equal to or lower than 110 mEq/L), as compared to NaCl 0.9%. Nonetheless, the final composition of such a fluid, especially when considering crystalloids, will depend on (1) tonicity; (2) electrical neutrality and (3) SID. Indeed, an isotonic balanced solution leaving unaltered acid–base equilibrium (i.e., with an SID close to 24 mEq/L) will necessarily have a Cl^−^ content > 110 mEq/L (as in Sterofundin-ISO). In contrast, a fluid with an SID of 24 mEq/L and a lower Cl^−^ content will necessarily be slightly hypotonic (as with Lactated Ringer’s). Finally, an isotonic fluid with a low Cl^−^ content will necessarily have a higher SID (as with PlasmaLyte), with a consequent alkalizing effect.

### The case for balanced solutions

Balanced and unbalanced (NaCl 0.9%) solutions might have slightly different effects on blood volume expansion, according to the clinical condition. Indeed, different kinetics showing approximately a 10% decrease in plasma volume expansion of balanced solutions as compared to NaCl 0.9% have been described in normovolemic healthy volunteers [25, 26]. On the other hand, in an experimental model of near-fatal hemorrhagic shock, a lower dose of balanced solution was needed, as compared to NaCl 0.9% to restore a target blood pressure [27]. These conflicting results underline the fact that findings about fluid therapy are condition-specific, and that results obtained from septic patients or experimental models should not be extrapolated to all situations.

Despite these controversies, which need further clarification, several definitive differences exist between these two categories of drugs. First, chloride-rich NaCl 0.9% causes a higher dose-dependent degree of acidosis and hyperchloremia, which possibly favors the contraction of vascular smooth muscles [28, 29], potentially leading to a reduced renal perfusion.

When healthy volunteers received 2 L of either saline or Plasma-Lyte over 1 h, saline significantly decreased renal artery blood velocity, decreased renal cortical tissue perfusion, decreased urine output, and increased extravascular fluid accumulation compared with Plasma-Lyte [30]. These findings may support the idea that hyperchloremia may cause increased tubule-glomerular feedback and decreased renal cortical perfusion [31].

Indeed, a large-scale propensity-matched observational analysis of U.S. insurance data showed that the use of PlasmaLyte^®^ versus NaCl 0.9% on the first day of major abdominal surgery led to significantly less renal failure requiring dialysis [32]. In addition to the effect on renal perfusion, NaCl 0.9%, being slightly hypertonic, likely causes an increased incretion of arginine vasopressin. These two effects can conceivably contribute to the slower renal excretion of NaCl 0.9% as compared to balanced solutions [33, 34]. Indeed, more fluid will be retained in the interstitial space, with the consequent propensity to cause more edema [35, 36]. However, it is not merely the renal function that could be deranged by high chloride concentrations; infusion of NaCl 0.9% can cause abdominal discomfort in healthy volunteers [37] and a reduced gastric perfusion in elderly surgical patients [38].

Two important and large randomized controlled trials comparing the use of balanced solutions and normal saline have been published in the last years. The SPLIT study was the first multi-center double-blind randomized controlled trial performed on 2092 patients, comparing balanced and unbalanced fluids in intensive care units. It showed no significant difference in the main outcome, i.e., incidence of acute kidney injury [39]. While providing a high level of evidence, this trial did not give a definitive answer. Indeed, the median volume of study fluid was only 2 L over 90 days. Moreover, both study groups had received a median volume of 1.0–1.2 L of PlasmaLyte within 24 h prior to enrolment, therefore making it plausible that prior administration of PlasmaLyte counterbalanced the effects of low-dose NaCl 0.9%. The SMART-trial was a large study performed in five intensive care units of a single academic center [40]. A total of 15,802 patients were randomized to receive either NaCl 0.9% or a balanced solution (Plasma-Lyte A or Lactated Ringer’s). In both groups, patients received an extremely small amount of fluids: a median of 1 L from admission to day 30 or discharge, whichever came first. Despite the unexpectedly low volume of crystalloids, the authors found a small difference in the primary outcome, i.e., the incidence of major adverse kidney events within 30 days (composite of death, new renal replacement therapy or persistent renal dysfunction) in favor of balance solutions. Looking at the overall outcome, it is important to emphasize that there was no reduction of in-hospital mortality and that neither the incidence of renal replacement therapy (2.5% vs. 2.9%, *p *= 0.08) nor the incidence of persistent renal dysfunction (6.4% vs. 6.6%, *p *= 0.60) was statistically significant. A similar study performed by the same authors and published in the same issue of the *New England Journal of Medicine*, the SALT-ED trial, found a similar difference in the incidence of major adverse kidney events in non-critically ill adults [41].

In summary, we can avoid fluid-induced metabolic acidosis and excessive chloride loading simply using balanced solutions. There is increasing evidence that an excessive chloride administration may have a detrimental effect on renal function, even at low doses. Therefore, the use of balanced solutions, particularly in patients that potentially need a significant amount of intravenous fluids, seems to be a reasonable pragmatic choice [42]. On the contrary, saline may be an intuitive choice for patients with hypovolemic hyponatremia or hypochloremic metabolic alkalosis. In any other settings, the most important reason to choose NaCl 0.9% over balanced solutions is likely economic in nature. Therefore, the patient’s serum chloremia is an important factor to determine the appropriate type of fluids.

## Albumin

### The basics

Albumin accounts for approximately 50% of the plasma protein content [43] and is the main determinant of plasma oncotic pressure, playing a crucial role in the regulation of microvascular fluid dynamics [44, 45]. Normal plasma concentration of albumin ranges between 35 and 55 g/L, corresponding to approximately 0.54–0.85 mmol/L, and to an in vitro pressure of approximately 9.2 mmHg. In contrast, in vivo colloid-oncotic pressure is lower, since the permeability of the endothelial barrier to albumin is variable, even in healthy subjects. Nonetheless, according to Starling’s law, oncotic pressure is the force counteracting intravascular hydrostatic pressure, therefore acting to reabsorb water and small solutes from the interstitium to the intravascular space. The crucial role of albumin’s oncotic property in the regulation of microcirculatory fluid dynamics also seems to apply to the endothelial glycocalyx layer [46, 47]. This gel-like layer, lining the luminal side of the endothelium, is thought to comprise 20% of the intravascular volume. The current view of the glycocalyx is that it holds many compounds that are mandatory for the functioning of the endothelium and mediates several key physiological processes, such as maintaining the vascular barrier, hemostasis, prevention of cell adhesion to the endothelium and transmission of shear stress [48]. The role of the glycocalyx is however under continuous investigation and its role and function might need to be revised in the future [49]. Of note, shedding of the glycocalyx occurs in the presence of reactive oxygen species, hyperglycemia, cytokines, and endotoxin, and is therefore common in critically ill patients [50]. In the context of fluid homeostasis, loss of barrier function induced by glycocalyx shedding is associated with the formation of edema [51]. Furthermore, fluid therapy itself is known to be potentially deleterious for endothelial function [27], likely because of the resulting oxidative stress. However, the risks probably relate to the specific clinical context. Indeed, while volume loading did not cause glycocalyx shedding in surgical patients and healthy volunteers [52, 53], the amount of glycocalyx shedding was proportional to the volume of fluid given in septic shock patients [54].

### The case for albumin

The ALBIOS study, a large Italian randomized controlled trial, gave some suggestions on whether or not albumin administration improves outcomes in severe sepsis and septic shock [55]. Patients with severe sepsis were randomized to receive either 20% albumin and crystalloids or crystalloids alone after initial early goal-directed resuscitation. In patients randomized to albumin treatment, albumin was supplemented for 28 days, to maintain an albumin concentration ≥ 30 g/L. Despite some beneficial physiologic effects (lower heart rates, higher mean arterial pressure, and lower daily net positive fluid balance over the first 7 days), no difference was observed either in mortality at 90 days (41.1% vs. 43.6%) or in overall organ failure scores. However, when analyzing the results according to disease severity, patients with septic shock randomized to albumin supplementation showed a lower risk of death (relative risk 0.87; 95% confidence interval—CI 0.77–0.99) as compared to those just receiving only crystalloids. It is worth mentioning that this trial did not utilize albumin as a resuscitation fluid, but as a drug to correct hypoalbuminemia.

### The case against albumin

Colloids should remain in the intravascular space longer than crystalloids, provided that the endothelial barrier is intact, which is often not the case in critically ill patients [56]. Given the recent discussion on the potential adverse effects of artificial colloids, especially of hydroxyethyl starches (HES), a renewed interest in the use of albumin has emerged. However, despite the strong physiologic rationale and significant scientific effort [55, 57], to date, no randomized controlled trial has shown any significant benefit of fluid resuscitation using albumin over other types of fluids, including crystalloids [58]. Some reports have even suggested that albumin administration in the setting of cardiac surgery may be associated with the development of acute kidney injury [59]. As stated previously, one of the largest albumin trials to date, the ALBIOS study, reported a reduction in 90-day mortality in a subgroup of patients with septic shock. However, this result was based on a post-hoc rather than predefined analysis and should, therefore, be interpreted with caution. The results of two ongoing randomized trials, the ALBumin Italian Outcome Septic Shock-BALANCED Trial (ALBIOSS-BALANCED) and the Albumin Replacement Therapy in Septic Shock (ARISS), may provide some answers to the above-mentioned issues.

The significant cost and the availability of equally effective low-cost alternatives do not play in favor of albumin, although a subgroup analysis of the ALBIOS dataset may suggest that albumin infusion is likely cost-effective in patients’ septic shock [60]. Up to date, the theoretical benefits of albumin are not supported by sound clinical evidence, and the case for albumin remains controversial.

## Perioperative fluid management

The aim of perioperative fluid therapy, in parallel with the maintenance of the effective circulating blood volume, is to avoid both fluid overload and under-hydration, while maintaining patients’ fluid balance as close as possible to zero. Despite this rationale, it is not unusual for surgical patients to receive 5–10 L of fluid and 600–1000 mmol of sodium, leading to edema and adverse outcomes [61], which is also favored by the marked and mean arterial pressure-dependent reduction of the elimination capacity of crystalloids [62, 63]. On the other hand, overnight fasting and bowel preparation, when traditionally applied, lead to fluid deficits. Apparently, patients develop postoperative complications when fluid retention exceeds 2.5 L [32, 64]. Of course, fluid gain depends not only on the amount of fluid administered, but also on the capacity of the kidney to excrete the excessive fluid and salt [32].

### Fluid management before surgery

Fluid therapy is not only meant to compensate intraoperative losses but should also take into account those occurring prior to surgery, induced by poor water intake, bowel preparation, major inflammation associated with a stress response, and possibly, hemorrhage. Dehydration, however, is difficult to detect through clinical methods.

Many studies examined whether a fluid load is capable of reducing hypotension caused by the induction of general/regional anesthesia. However, results regarding a preload strategy have been discouraging [65, 66].

### Fluid management during surgery

In response to the ongoing administration of large volumes of crystalloid to patients undergoing major surgery, a ‘fluid restrictive’ strategy has been proposed. For example, Brandstrup et al. demonstrated in a multi-center randomized controlled trial that a more restrictive regimen was associated with better outcomes following colorectal surgery [61]. However, the regimen was *restrictive* compared with the standard of care that was *excessive* (e.g., 5 L positive balance due to high crystalloid volumes) [67]. It is therefore conceivable that the group with a better outcome rather benefitted from the avoidance of fluid excess than from fluid restriction. The interpretation of the literature on the topic is hampered by the use of very heterogeneous definitions [68]. What is however clear from observational studies is that both too much and too little fluid are associated with poor outcomes [69–72]. Recently, a large cohort study from 500 U.S. hospitals including adult patients having colon, rectal or primary hip or knee surgery was concluded [72]. A significant association was found between liberal fluid administration on the day of surgery and worse outcomes (increased total costs and length of stay in all patients), as well as increased presence of postoperative ileus, in patients undergoing colorectal surgery. Interestingly, the authors also observed that restrictive fluid utilization (the lowest 25% by volume) was also associated with worse outcomes.

It is common in Enhanced Recovery after Surgery (ERAS) protocols to find the term “intraoperative fluid restriction” [73]. However, alternative terms, such as “zero balance” or the avoidance of salt and water excess, are also available. Protocols advocate the infusion of balanced crystalloid of 1–3 ml/kg/h and to give additional boluses of fluid only to match needs judged by either measured volumes lost during surgery, or the assessment of peripheral perfusion (such as according to the so-called ‘Goal-Directed Fluid Restriction’) [74]. Overall, the literature suggests that algorithm-based perioperative fluid regimens result in improved patient outcomes.

### Fluid management after surgery

Fluid management in postoperative patients is a key determinant of their outcomes. While restoring effective volume is critical for these patients, fluid management should not compromise healing processes. Optimal fluid management should thus target efficient central hemodynamics and tissue perfusion while avoiding positive net fluid balance. In theory, colloids offer the advantages over crystalloids of higher plasma expansion capacity and longer plasma half-life. They have the theoretical disadvantage of delaying clotting time and increasing the risk of kidney injury. In randomized trials, the ratio of the cumulative dose of colloids over the cumulative dose of crystalloids ranged roughly from 0.41 to 1 [75]. In patients with overt clinical hypovolemia, colloids were superior to crystalloids in improving cardiac filling pressures and performance [76]. Likewise, in a large multinational randomized trial performed in critically ill patients with acute hypovolemia, colloids reduced vasopressor and ventilator dependency when compared to crystalloids [77]. A recent systematic review of resuscitation with HES in surgical critically ill patients identified 13 randomized trials [78]. However, this review found no statistically significant difference between HES and crystalloids, in terms of mortality (risk ratio 2.97; 95% CI 0.96 to 9.19; *I*_2_  =  0%), need for renal replacement therapy (risk ratio 1.11; 95% CI 0.26 to 4.69; *I*_2_  =  34%), and major infectious complications (risk ratio 1.19; 95% CI 0.59 to 2.39; *I*_2_ =  0%). It is worth mentioning that eligible trials were too small to draw firm conclusions on this issue.

It should also be stated that there are opposing views regarding the use of starches [79]. For example, several criticisms regarding the CHEST trial have been put forward which still require to be addressed [80, 81]. Furthermore, it can be stated that in the CHEST trial starches were administered to patients that were not hypovolemic. On the other hand, the CRISTAL trial (where 70% of the colloid group received HES) concluded that significantly less volume was required to achieve hemodynamic stability for HES vs. NaCl in the initial phase of fluid resuscitation in severe sepsis patients without any difference for adverse events in both groups [77]. Taking these opposing views into consideration, the ongoing debate about the use of starches in hypovolemic critically ill patients still requires more data.

Among patients undergoing major abdominal surgery, the recent results of the FLASH trial, showed no significant difference in a composite outcome of death or major postoperative complications within 14 days after surgery [82].

Pending the results of ongoing trials, there are currently insufficient data to ban the use of colloids in the surgical intensive care unit.

Many patients undergoing surgery are not able to ingest food or fluids for some time following surgery and will require maintenance fluids. Recently, a debate emerged on the tonicity of these solutions: although guidelines traditionally recommended the use of hypotonic maintenance fluids, in pediatric literature, these were shown to be associated with an increased incidence of symptomatic hyponatremia [83, 84]. The recent randomized controlled TOPMAST trial in adults undergoing major thoracic surgery found this problem to be mild in these patients. Isotonic maintenance fluids, on the other hand, were associated with a considerably larger positive cumulative fluid balance (estimated at 1.4L more positive under fluids containing 154 compared to 54 mmol/L of sodium) [85].

## Fluid overload

### The problem with fluid overload in the perioperative setting

A certain degree of hypervolemia is necessary to maintain organ perfusion during anesthesia and surgery. However, fluid given after the induction of anesthesia mainly increases “unstressed” blood volume, because vasodilatation occurs as a consequence of anesthesia. At this point, additional fluid administration is needed to optimize stroke volume, i.e., to add to the “stressed” intravascular volume [86]. Many clinicians still consider this “wet” approach as the gold standard for intraoperative fluid therapy [87], although intravascular volume expansion certainly bears some dangers. Myocardial work and cardiac pressures increase when infused fluids have exceeded the degree of anesthesia-induced vasodilatation. Moreover, fluid overload reduces the colloid osmotic pressure that, together with raised cardiac pressures, might promote pulmonary edema [88]. These issues are of particular relevance in patients with poor cardiovascular status. Finally, hypervolemia may be responsible for another important effect: the release of atrial natriuretic peptides (ANPs) to the circulation caused by the stretching of atrial myocardial fibers [68, 89]. Indeed, in response to a rapid infusion of crystalloids, ANP levels increase 2- to 3-fold [90–92], therefore reducing strain on the circulation by promoting natriuresis and capillary leakage of albumin.

### The problem with fluid overload in the Intensive Care Unit

Fluid administration is one of the cornerstones of hemodynamic resuscitation in critically ill patients. How much fluid to give has been the subject of lively debate over the years. Too much fluid can have harmful consequences on multiple organ systems, e.g., worsening gas exchange, renal function and wound healing. Fluid overload is particularly likely to arise in conditions when capillary permeability is altered due to an inflammatory response, such as during sepsis. A positive fluid balance has been associated with worse outcomes in several studies in various groups of intensive care unit (ICU) patients [16, 93–95]. In patients with septic shock, fluid administration and positive fluid balance were independently associated with increased mortality rates [93, 96]. Similarly, in patients admitted to the ICU after major surgery, fluid balance was an independent risk factor for death [95]. Indeed, a multi-modal restrictive fluid strategy aiming for negative fluid balance (PAL-treatment) in patients with acute lung injury (ALI) was associated with improved outcomes in a retrospective study [97].

It has to be acknowledged that a positive fluid balance could be a marker of disease rather than a pure iatrogenic or preventable problem and it would be erroneous to assume the default position of under-resuscitation. Indeed, inadequate resuscitation due to insufficient fluid administration may result in poorer tissue perfusion and hence organ dysfunction and failure, particularly in the early phase of treatment. A balance needs to be achieved, such that each patient receives sufficient, but not excessive, fluid for her/his needs. Crucially, different patients will have different needs and baseline fluid status depending on multiple factors including age, co-morbid disease and current diagnosis. In addition, it is mandatory to consider indices of fluid tolerance, such as CVP, lung water, oxygenation and hemoglobin levels. Fluid requirements vary during the course of illness. As such, fluids must be prescribed on an individual patient basis; the prescription should be regularly reviewed and tailored to the evolving clinical stage. The answer to the question of whether fluid overload is a problem in the ICU will thus depend on when it is asked. In the acute resuscitation/salvage phase, fluid administration is generous. While fluid overload should always be a concern, a positive fluid balance is a specific target of this phase.

### Is deresuscitation/de-escalation the solution?

The term deresuscitation/de-escalation was first suggested in 2012 [98] and finally coined in 2014 [16]. It specifically refers to ‘Late Goal-Directed Fluid Removal’, which involves “aggressive and active fluid removal through diuretics and renal replacement therapy with net ultrafiltration”. Deresuscitation/de-escalation is sometimes also used to more loosely refer to the phase of critical illness and/or the care of a critically ill patient, after initial resuscitation, stabilization, and optimization. It is characterized by the discontinuation of invasive therapies and a reduction of a spurious fluid balance. Late conservative fluid management is defined as 2 consecutive days of negative fluid balance within the first week of ICU stay, and is an independent predictor of survival in ICU patients [16].

Fluid overload and a positive cumulative fluid balance are associated with increased morbidity and worse outcomes, as previously discussed. The natural course of events after a first insult (such as infection, trauma, etc.) is a systemic inflammatory response with increased capillary permeability and organ dysfunction [98]. The presence of fluid overload and interstitial edema may thus trigger a vicious cycle. This is what has been referred to as the *Ebb* phase of shock [16]. In the majority of patients, shock reversal occurs (with correct antibiotics and proper source control) and excess fluids can be mobilized: this is called the *Flow* phase [16]. However, some patients will not transfer spontaneously from the *Ebb* to *Flow* phase and will remain in a state of unresolved shock with positive cumulative fluid balance, and this is where active deresuscitation/de-escalation might have an important role.

It is unclear which is the best therapeutic option for deresuscitation/de-escalation. The administration of albumin in combination with diuretics (20% albumin to achieve a serum albumin levels of 30 g/L and furosemide bolus of 60 mg followed by continuous infusion of 10 mg/h) and the association of this strategy with the sequential application of PEEP set to counteract intra-abdominal pressure (IAP) have been proposed [97]. In addition, renal replacement therapy and aggressive ultrafiltration can be used to achieve a negative fluid balance in selected patients [99]. When it comes to deresuscitation/de-escalation, it is important to decide on when, how and for how long. For this purpose, we need to use the right targets to reach our goals. “Over-deresuscitation” has its drawbacks and may cause neurologic dysfunction in the long run [100].

In conclusion, it is crucial to ensure that the indication for fluid resuscitation no longer exists (e.g., absence of vasopressor, no lactate, adequate venous oxygen saturation of hemoglobin) before starting with deresuscitation. Furthermore, the *5 steps of Deresuscitation*/*De*-*Escalation* need to be kept in mind: (1) define a clinical endpoint (e.g., improvement in oxygenation); (2) set a fluid balance goal (e.g., 1 L negative balance in 24 h); (3) set perfusion and renal safety precautions (e.g., vasopressor need, 25% serum creatinine increase); (4) re-evaluate after 24 h unless safety limits reached; (5) adjust the plan accordingly.

## The 4 phases of fluid therapy and the R.O.S.E. or S.O.S.D. concept

**Fig. 1:**
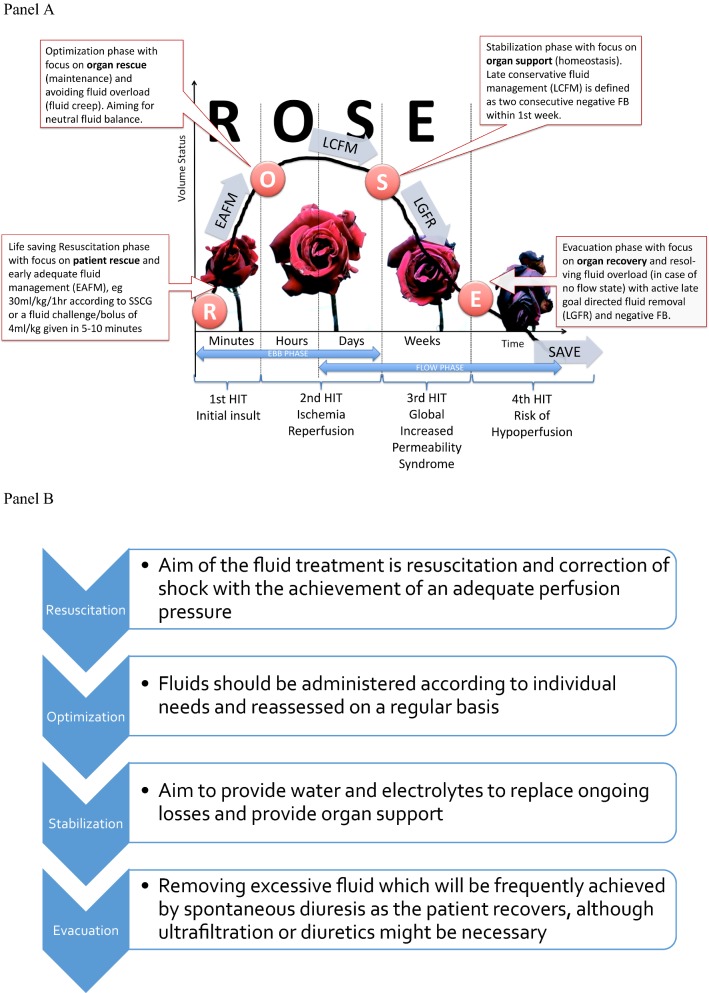
The R.O.S.E. concept and the 4 phases of Fluid Therapy. Adapted according to the Open Access CC BY Licence 4.0 with permission from Malbrain et al. [9]. **a** Graph showing the four-hit model of shock with evolution of patients’ cumulative fluid volume status over time during the five distinct phases of resuscitation: Resuscitation (R), Optimization (O), Stabilization (S), and Evacuation (E) (ROSE), followed by a possible risk of Hypoperfusion in case of too aggressive deresuscitation. On admission patients are hypovolemic, followed by normovolemia after fluid resuscitation (EAFM, early adequate fluid management), and possible fluid overload, again followed by a phase going to normovolemia with late conservative fluid management (LCFM) and late goal-directed fluid removal (LGFR) or deresuscitation. In case of hypovolemia, O_2_ cannot get into the tissues because of convective problems, in case of hypervolemia O_2_ cannot get into the tissue because of diffuse problems related to interstitial and pulmonary edema, gut edema (ileus and abdominal hypertension). **b** The role of fluids within the R.O.S.E. concept

Two articles were published recently, almost simultaneously, referring to the dynamics of fluid therapy [16, 101]. These conceptual models identified *four* dynamic phases. The Acute Dialysis Quality Initiative (ADQI) group proposed S.O.S.D. (Salvage, Optimization, Stabilization, De-escalation) as acronym [101]. However, during the *International Fluid Academy Day* (IFAD) meetings there was a clear preference for the R.O.S.E. acronym (Resuscitation, Optimization, Stabilization, Evacuation) as summarized below, in Fig. 1 and Table 3. We tried to suggest endpoints and targets for the different phases; however, it was decided not to include them because there cannot be a specific target of cardiac index and PPV must be considered only if cardiac output is low. A high PPV is often a physiological state and defining a “normal” state when a low PPV value is reached might lead to unnecessary fluid infusion [102]. Also, defining a given preload level as a target of resuscitation is senseless as it may shift from patient to patient and from time to time.

**Table 3:** The 4 dynamic phases of fluid therapy according to the ROSE concept. Adapted from Malbrain et al. [4] with permission

	Resuscitation (R)	Optimization (O)	Stabilization (S)	Evacuation (E)	
HIT	First	Second	Second	Third	Fourth
Cause	Inflammatory insult, e.g., sepsis, severe acute pancreatitis (SAP), burns, trauma, etc.	Ischemia and reperfusion	Ischemia and reperfusion	GIPS (global increased permeability syndrome)	Hypoperfusion
Phase	Ebb	Flow	Flow/no flow	No flow	No flow
Type	Severe shock	Unstable	Stable	Recovering	Unstable
Example	Septic shock, major trauma, hemorrhagic shock, ruptured abdominal aortic aneurysm, severe acute pancreatitis, severe burns (> 25% TBSA)	Intra- and perioperative goal-directed therapy, less severe burns (< 25% TBSA), diabetic keto-acidosis, severe gastro-intestinal losses (vomiting, gastroenteritis)	Postoperative patient (nil per mouth or combination of total enteral plus parenteral nutrition), abdominal vacuum-assisted closure, replacement of losses in less-severe pancreatitis	Patient on full enteral feed in recovery phase of critical illness, polyuric phase after recovering from acute tubular necrosis	Patient with cirrhosis and anasarca edema (GIPS) and no Flow state, hepatosplanchnic hypoperfusion
Question	When to start fluids?	When to stop fluids?	When to stop fluids?	When to start unloading?	When to stop unloading?
Subquestion	Benefits of fluids?	Risks of fluids?	Risks of fluids?	Benefits of unloading?	Risks of unloading?
O_2_ transport	Convective problems	Euvolemia, normal diffusion	Diffusion problems	Euvolemia, normal diffusion	Convective problems
Fluids	Mandatory	Biomarker of critical illness	Biomarker of critical illness	Toxic	
Fluid therapy	Rapid bolus (4 ml/kg/10–15 min)	Titrate maintenance fluids, conservative use of fluid bolus	Minimal maintenance if oral intake inadequate, provide replacement fluids	Oral intake if possible Avoid unnecessary IV fluids	Avoid hypoperfusion
Fluid balance	Positive	Neutral	Neutral/negative	Negative	Neutral
Result	Life saving (rescue, salvage)	Organ rescue (maintenance)	Organ support (homeostasis)	Organ recovery (removal)	Organ support
Targets	Macrohemodynamics (MAP, CO); lactate; volumetric preload (LVEDAI); functional hemodynamics; fluid responsiveness (PLR, EEO)	Organ macroperfusion (MAP, APP, CO, ScvO_2_); volumetric preload (GEDVI, RVEDVI); GEF correction; R/L shunt; think of polycompartment syndrome, CARS	Organ function (EVLWI, PVPI, IAP, APP); biomarkers (NGAL, cystatin-C, citrullin); capillary leak markers (colloid oncotic pressure, osmolality, CLI, RLI); daily and cumulative FB, body weight	Organ function evolution (P/F ratio, EVLWI, IAP, APP, PVPI) Body composition (ECW, ICW, TBW, VE)	Organ microperfusion (pHi, ScvO_2_, lactate, ICG-PDR); Biomarkers; Negative cumulative fluid balance
Monitoring tools	Arterial-line, central venous-line, PPV or SVV (manual or via monitor), uncalibrated CO, TTE, TEE	Calibrated CO (TPTD, PAC)	Calibrated CO (TPTD); Balance; BIA (ECW, ICW, TBW, VE)	Calibrated CO (TPTD); balance; BIA; DE-escalation	LiMON, Gastric tonometry, micro-dialysis
Goals	Correct shock (EAFM—early adequate fluid management)	Maintain tissue perfusion	Aim for zero or negative fluid balance (LCFM—late conservative fluid management)	Mobilize fluid accumulation (LGFR—late goal-directed fluid removal = emptying) or DE-resuscitation	Maintain tissue perfusion
Timeframe	Minutes	Hours	Days	Days to weeks	Weeks

*APP* abdominal perfusion pressure, = MAP − IAP, *BIA* bio-electrical impedance analysis, *CARS* cardio-abdominal renal syndrome, *CLI* capillary leak index, = serum CRP divided by serum albumin, *CO* cardiac output, *ECW* extracellular water, *EEO* end-expiratory occlusion test, *EVLWI* extravascular lung water index, *GEDVI* global end-diastolic volume index, *GEF* global ejection fraction, *GIPS* global increased permeability syndrome, *IAP* intra-abdominal pressure, *ICG-PDR* indocyaninegreen plasma disappearance rate, *ICW* intracellular water, *IV* intravenous, *LVEDAI* left ventricular end-diastolic area index, *MAP* mean arterial pressure, *P/F* pO_2_ over FiO_2_ ratio, *PLRT* passive leg raising, *PPV* pulse pressure variation, *PVPI* pulmonary vascular permeability index, *RLI* renal leak index, = urine albumin divide by urine creatinine, *R/L* right to left shunt, *RVEDVI* right ventricular enddiastolic volume index, *SAP* severe acute pancreatitis, *ScvO*_*2*_ central venous oxygen saturation, *SVV* stroke volume variation, *TBSA* total burned surface area, *TBW* total body water, *TEE* transesophageal echocardiography, *TPTD* transpulmonary thermodilution, *TTE* transthoracic echocardiograph, *VE* volume excess

### Resuscitation phase (R) or salvage phase (S)

In the first, salvage/resuscitation phase, when a patient presents with hemodynamic shock, the aim of the treatment is resuscitation and correction of shock with the achievement of an adequate perfusion pressure. A rapid fluid bolus should be given (although the exact amount can vary, usually 3–4 mL/kg given over 10 to 15 min and repeated when necessary), normally in association with vasopressor administration. In parallel, emergency procedures to resolve any obvious underlying cause should be performed, with hemodynamic monitoring initiated. In this phase, the goal is early adequate goal-directed fluid management: fluid balance must be *positive*. We do not support blind adherence to the surviving sepsis campaign guidelines adagio to administer 30 ml/kg of fluids within the first hour for all patients, as explained previously [9]. This may lead to either over- or under resuscitation in some patients. Every patient needs an individual and personalized approach.

### Optimization phase (O)

**Fig. 2:**
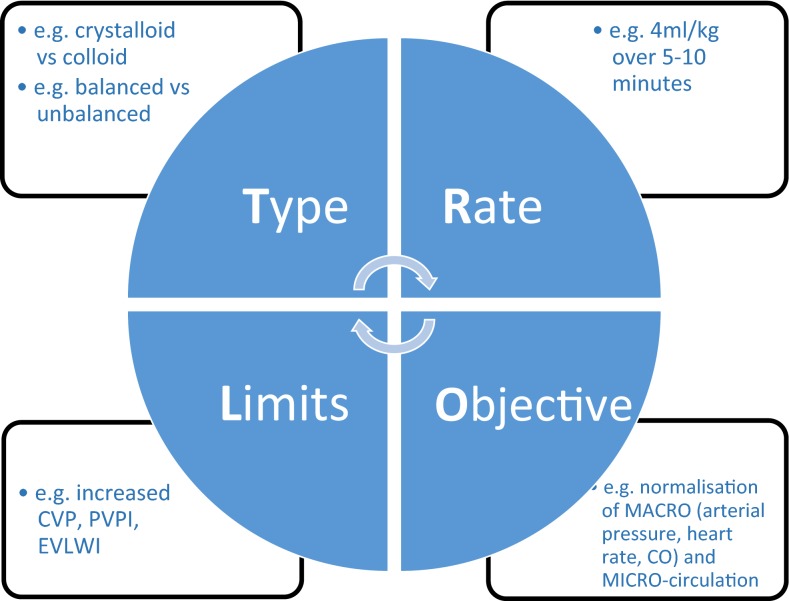
The TROL mnemonic of fluid challenge: considerations for administration of a fluid bolus in critically ill patients. *CO* cardiac output; *CVP* central venous pressure; *EVLWI* extra vascular lung water index; *PVPI* pulmonary vascular permeability index (Adapted from Vincent and Weil [97])

The optimization phase starts when the patient is no longer in overt absolute/relative hypovolemia, but remains hemodynamically unstable. Some form of monitoring will by now be in place. Fluids should be administered according to individual needs, reassessed on a regular basis, e.g., using fluid challenge techniques [103, 104]. Fluid challenges must be conducted carefully, bearing in mind the four essential components (TROL): Type of fluid (e.g., a balanced crystalloid-like PlasmaLyte); Rate (e100–200 mL over 10 min); Objective (e.g., normal arterial pressure or heart rate); and Limits (e.g., high central venous pressure level) (Fig. 2) [105]. The aim of this phase is to optimize and maintain adequate tissue perfusion and oxygenation in order to prevent and limit organ damage. The patient must be carefully monitored during the optimization phase: often several types of monitoring (e.g., arterial catheter, echocardiography, central venous pressure, arteriovenous blood gas) are required to obtain the most complete picture of a patient’s hemodynamic status.

Although a resuscitation based on microcirculatory endpoints is expected to result in analogous amelioration in the microcirculation, a lack of coherence may exist between macro- and microcirculation. Thus, markers of hypoperfusion should include also lactate, prolonged capillary refill time and mottling score [106].

### Stabilization phase (S)

Once the patient is stable, the stabilization phase begins and evolves over days. In this phase, the aim of fluid management is to ensure water and electrolytes to replace ongoing losses and provide organ support. The target should be a zero or slightly negative fluid balance. It might be of interest, in this context, to underline the fact that in the major trials suggesting a harmful effect of starches [2, 107], these colloids were given abundantly also in the stabilization phase, i.e., in a phase that possibly did not require these drugs.

### Evacuation phase (E) or de-escalation phase (D)

The final phase is evacuation or de-escalation, with the purpose of removing excessive fluid. This will be frequently achieved by spontaneous diuresis as the patient recovers, although ultrafiltration or diuretics might be necessary. Of note, it was recently shown that diuretics might favor the recruitment of microcirculation, thus decreasing diffusion distances and improving oxygen extraction [108].

## Fluid management in trauma and burns

### Fluid management in acute hemorrhagic shock following trauma

Although traumatic brain injury remains the commonest cause of death following severe blunt injury, concomitant major hemorrhage will result in cerebral hypoperfusion, which undoubtedly contributes to secondary brain injury and death. As such, hemorrhage remains the most preventable cause of trauma mortality.

An adequate intravascular volume, hemoglobin concentration and oxygen saturation are essential to maintain aerobic metabolism. Humans do not tolerate anaerobic metabolism and 90% of oxygen consumption is used in the formation of adenosine triphosphate (ATP), the major energy source for cell function. Rapid reversal of anaerobic metabolism is imperative to restore ATP and prevent irreversible cellular apoptosis and death [109].

Recognizing that hypovolemia is the consequence of hemorrhagic shock, past strategies utilized crystalloids to restore intravascular volume, followed by blood transfusion. Crystalloids, however, do not carry oxygen, and oxygen delivery may only be enhanced by an adequate hemoglobin concentration. Furthermore, major hemorrhage is accompanied by a unique coagulation disorder, the Acute Coagulopathy of Trauma and Shock (ACoTS) [110], leading to poor clot formation, as a result of increased binding of thrombin to thrombomodulin and enhanced fibrinolysis. Dilution of coagulation factors, acidosis, and hypothermia play a secondary role in this scenario. The approach to resuscitation must therefore be proactive and not reactive with the combined administration of packed red blood cells, plasma, platelets, and cryoprecipitate. The use of clear resuscitation fluids should be minimized. Based on military experience, the recommended ratio of packed red blood cells to plasma and platelets should be 1:1:1. The endpoints for hemoglobin concentration of 10 g/dL, a platelet count of > 50,000, an INR < 1.5 and a fibrinogen concentration of > 1 g/L cannot be generally recommended. In addition, the ionized calcium level should be > 1.0 mmol/L.

While the above is a general recommendation, not all patients will require such an aggressive approach [111]. Indeed, over-zealous transfusion is associated with unwanted complications.

The standard approach has been to use conventional laboratory coagulation testing to determine the need for component therapy. These, however, are performed at room temperature and do not reflect individual steps in coagulation. Thromboelastometry has now been recognized as an essential tool to monitor coagulopathy in trauma [112]. This device reflects the entire process of coagulation and can graphically determine the need for specific coagulation factors. Unlike laboratory coagulation studies, modern thromboelastometry machines may be set to the patient’s core temperature and accurately reflect the in vivo coagulation status. These instruments should be the standard of care in centers handling major trauma.

Following the CRASH-2 trial indicating the benefit of tranexamic acid given within 3 h from injury, such treatment has been included in many protocols for major hemorrhage [112]. In the presence of a sophisticated trauma system, the benefits are doubtful and further data are warranted [113].

### Fluid management in burns

The understanding of burn shock pathophysiology and subsequent development of fluid resuscitation strategies resulted in dramatic outcome improvements in burn care during the last decades [114]. However, while under-resuscitation has become rare in clinical practice, there is growing concern that over-resuscitation, leading to increased morbidity and mortality, has become more of an issue in burn care. In the late sixties of the previous century, Baxter and Shires developed their landmark formula at the Parkland Memorial Hospital, which has lasted decades as the gold standard for fluid resuscitation in acute burn care across the world [115]. The formula advocates 4 ml crystalloids per kg per % of total body surface area for 24 h, of which half is given during the first 8 h. Diuresis (target 1 ml/kg/h) is used to guide the amount of intravenous fluids. During the second 24 h of resuscitation, colloids are allowed, and resuscitation volume is adapted according to diuresis (with a gradual decrease if diuresis is adequate).

However, over the last 15 years, multiple centers have reported excess fluid administration [116, 117]. This fluid excess often leads to “resuscitation morbidity”, a group of complications linked to fluid overload, such as delayed wound healing, delayed recovery of gastro-intestinal function (with ileus), pulmonary edema (due to capillary leak and increased extravascular lung water), limb compartment syndrome, orbital compartment syndrome, intra-abdominal hypertension and abdominal compartment syndrome leading to multiple organ failure [118–120].

This discrepancy between the predicted and the administered fluid is known as “fluid creep”, a term brought to life by Basil Pruitt [119].

Recommendations for fluid resuscitation in burns are listed in Table 4. The most well-known adverse effect of NaCl 0.9% is hyperchloremic metabolic acidosis. Given the large infusion volumes administered to burn patients, balanced solutions are preferred. Indeed, since the beginning of burn resuscitation, most formulae advocate the use of balanced crystalloid solutions. Of note, an observational study reported lower Sequential Organ Failure Assessment (SOFA) scores in severely burned patients resuscitated with acetated Ringer’s [121].

**Table 4:** Recommendations regarding fluid resuscitation in severe burns’ patients. Adapted from Peeters et al. [106] with permission

Type of fluid	Recommendation
1. Normal saline	Given the fact that fluid resuscitation in burn management requires large volumes, the use of saline cannot be recommended in a burn resuscitation protocol
2. Balanced crystalloid	Based on the available evidence, balanced crystalloid solutions are a pragmatic initial resuscitation fluid in the majority of acutely ill (and burn) patients
3. Semi-synthetic colloids	Given the recent data concerning the use of semi-synthetic colloids (and starches in particular), their use in critically ill patients, including burn patients, cannot be recommended
4. Albumin	Based on the available evidence the use of albumin 20% can be recommended in severe burns, especially in the deresuscitation phase guided by indices of capillary leak, body weight, (cumulative) fluid balance, fluid overload, extravascular lung water and IAP
5. Hypertonic solutions	To this day, there is insufficient evidence to reach consensus regarding the safety of hypertonic saline in burn resuscitation. Whenever using hypertonic saline in clinical practice, however, close monitoring of sodium levels is highly advised

The use of colloids in the first 24 h has been controversial since it was thought that the existing capillary leak would allow large molecules to leak out into the extravascular space and exert an osmotic pull increasing the formation of edema [122]. In the last 15 years, renewed interest in colloids has arisen during burn resuscitation, instigated by the awareness of morbidity related to resuscitation and fluid creep. Until recently, the low molecular weight HES solutions were widely used as a resuscitation fluid in critically ill ICU, surgery and burn patients. However, after large fluid trials, including the CHEST and 6S trials, showing increased mortality and a higher rate of renal replacement therapy have raised alarming conclusions regarding the safety of HES solutions, starches can no longer be used in burn injuries as recommended by the Pharmacovigilance Risk Assessment Committee (PRAC) [2, 107, 123].

Albumin is a natural plasma protein that contributes most to intravascular oncotic pressure in humans (see above). The most common solutions are 4%, 5% or 20% albumin. It is a relatively expensive solution and its availability may be limited in some countries. Although albumin resuscitation has been used with some reservations, especially in the acute phase of burn resuscitation, trials provide promising data regarding the use of albumin as an adjunctive therapy in burn resuscitation [124, 125]. Similarly, hypertonic saline has been used for decades in burn resuscitation; theoretically, it expands the circulating volume by an intravascular water shift. Proponents claim that this process will decrease tissue edema and will lower the rate of complications. This hypothesis, however, needs to be confirmed by further studies.

## Take home messages and considerations prior to IV fluid prescription

**Fig. 3:**
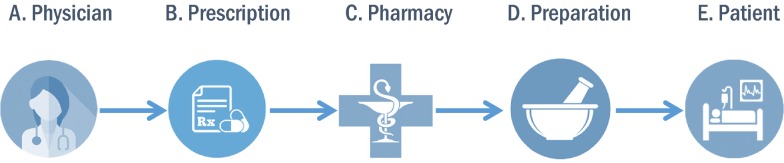
The 5 Ps of fluid administration. **a** Physician: All starts with the physician’s participation in making decisions related to fluid management. **b** Prescription: The physician should engage in writing a prescription that accounts for drug, dose, duration and whenever possible de-escalation. **c** Pharmacy: The prescription is sent to the pharmacy and is checked for inconsistencies by the pharmacist to get a more holistic view. **d** Preparation: The process by which the prescription is prepared and additions (e.g., electrolytes) made. **e** Patient: The filled prescription goes back to the patient and fluid stewards should observe administration, response, and debrief

Consider the 5 Ps of fluid prescription as shown in Fig. 3 and tailor the IV fluids to the patient’s need via individualized and personalized care (Table 5) [126]. Prescription safety can be summarized by the ‘4 Ds’ principle as explained above [4]:

**Table 5:** The four stages to check for appropriateness of IV fluid therapy. Adapted with permission from Malbrain ML et al. [126]

Stage of evaluation	Audit standard
1. Assessment	The patient’s fluid balance (via fluid chart with input and output) is assessed on admission in the hospital and on a day-by-day basis The patient’s weight is assessed within the last 3 days of fluid prescription The patient’s fluid and electrolyte needs are assessed as part of every ward review The assessment of the patient’s fluid status (hypo/eu/hypervolemia) includes the use of clinical judgement, vital signs and fluid balance with urine output Recent lab results with urea and electrolytes (within 24 h of fluid prescription) If possible sodium balance should be reported
2. Indication	A. Resuscitation For patients in need of fluid resuscitation: The cause of the fluid deficit is identified An assessment of shock or hypoperfusion was made A fluid bolus of 4 mL/kg of balanced crystalloids is given Fluid responsiveness is assessed with functional hemodynamics, passive leg raising test or end-expiratory occlusion test, or a combination Mean arterial pressure and cardiac output are monitored continuously via pulse contour analysis allowing assessment of beat-to-beat variations Patients who have received initial fluid resuscitation are reassessed within 30 min Care is upgraded in patients who have already been given > 2000 mL of crystalloids and still need fluid resuscitation after reassessment Patients who have not had > 2000 mL of crystalloids and who still need fluid resuscitation after reassessment receive 2–4 mL/kg of crystalloids and have a further reassessment
	B. Maintenance If patients need IV fluids for routine maintenance alone, the initial prescription is restricted to 25–30 mL/kg/day (1 mL/kg/h) of water and Approximately 1 mmol/kg/day of potassium (K^+^) and Approximately 1–1.5 mmol/kg/day of sodium (Na^+^) and Approximately 1 mmol/kg/day of chloride and Approximately 50–100 g/day (1–1.5 g/kg/day) of glucose to limit starvation ketosis Definition of inappropriateness in case of electrolyte disturbances Solutions not containing adequate amount of sodium in case of hyponatremia (Na < 135 mmol/L) Solutions not containing adequate amount of potassium in case of hypokalemia (K < 3.5 mmol/L) Solutions containing too much sodium in case of hypernatremia (Na > 145 mmol/L) Solutions containing too much potassium in case of hypokalemia (K > 5 mmol/L) The amount of fluid intake via other sources should be subtracted from the basic maintenance need of 1 ml/kg/h: Enteral or parenteral nutrition Fluid creep (see further)
	C. Replacement and redistribution If patients have ongoing abnormal losses or a complex redistribution problem, the fluid therapy is adjusted for all other sources of fluid and electrolyte losses (e.g., normal saline may be indicated in patients with metabolic alkalosis due to gastro-intestinal losses)
	D. Fluid creep All sources of fluids administered need to be detailed: crystalloids, colloids, blood products, enteral and parenteral nutritional products, and oral intake (water, tea, soup, etc.) Precise data on the concentrated electrolytes added to these fluids or administered separately need to be collected Fluid creep is defined as the sum of the volumes of these electrolytes, the small volumes to keep venous lines open (saline or glucose 5%), and the total volume used as a vehicle for medication
3. Prescription	The following information is included in the IV fluid prescription: The type of fluid The rate of fluid infusion The volume or dose of fluid The IV fluid prescription is adapted to current electrolyte disorders and other sources of fluid intake
4. Management	Patients have an IV fluid management plan, including a fluid and electrolyte prescription over the next 24 h The prescription for a maintenance IV fluid only changes after a clinical exam, a change in dietary intake or evaluation of laboratory results

- Drug—which fluid.
- Dose—calculate how much to give.
- Duration—duration of the IV fluid therapy.
- De-escalation—stop it as soon as possible.

The bottom line is *“Give the right fluid in the right dose to the right patient at the right time”*

## Conclusions

The prescription of fluid therapy is one of the most common medical acts in hospitalized patients but many of the aspects of this practice are surprisingly complex. It is time to introduce fluid stewardship in your ICU. To avoid fluid-induced harm, we recommend a careful evaluation of the chosen solution and a phase-wise approach to its administration, taking into account the clinical course of the disease or surgical procedure. Fluids should be prescribed with the same care as any other drug and every effort should be made to avoid their unnecessary administration.

## Data Availability

Not applicable.

## Abbreviations

4 Ds: Drug—Dose—Duration—De-escalation
ANP: Atrial natriuretic peptide
ATP: Adenosine triphosphate
CI: Confidence interval
ERAS: Enhanced recovery after surgery
HES: Hydroxyethyl starch
IAP: Intra-abdominal pressure
ICU: Intensive care unit
PEEP: Positive end-expiratory pressure
ROSE: Resuscitation—Optimization—Stabilization–Evacuation
SID: Strong ion difference
SOFA: Sequential Organ Failure Assessment
